# Recent advances in covalent, site-specific protein immobilization

**DOI:** 10.12688/f1000research.9002.1

**Published:** 2016-09-12

**Authors:** Morten Meldal, Sanne Schoffelen

**Affiliations:** 1 Center for Evolutionary Chemical Biology, Department of Chemistry & Nano-Science Center, University of Copenhagen, Copenhagen, Denmark

**Keywords:** enzyme-mediated immobilization, self-labeling protein tag, bioorthagonal reactions, oxime ligation, azide-alkyne cycloaddition

## Abstract

The properties of biosensors, biomedical implants, and other materials based on immobilized proteins greatly depend on the method employed to couple the protein molecules to their solid support. Covalent, site-specific immobilization strategies are robust and can provide the level of control that is desired in this kind of application. Recent advances include the use of enzymes, such as sortase A, to couple proteins in a site-specific manner to materials such as microbeads, glass, and hydrogels. Also, self-labeling tags such as the SNAP-tag can be employed. Last but not least, chemical approaches based on bioorthogonal reactions, like the azide–alkyne cycloaddition, have proven to be powerful tools. The lack of comparative studies and quantitative analysis of these immobilization methods hampers the selection process of the optimal strategy for a given application. However, besides immobilization efficiency, the freedom in selecting the site of conjugation and the size of the conjugation tag and the researcher’s expertise regarding molecular biology and/or chemical techniques will be determining factors in this regard.

## Introduction

**Figure 1.**
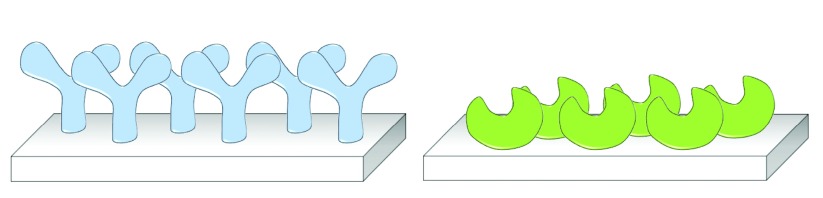
Schematic representation of oriented immobilization of (left) antibodies and (right) biocatalysts. Control over the site of immobilization ensures maximum accessibility to the analyte binding site and the biocatalyst’s active site.

Protein immobilization plays an important role in the fields of life science and medicine. It forms the basis for many bio-based applications involving protein–protein or protein–ligand interactions, such as biosensors, biomedical implants, recyclable biocatalysts, and protein arrays for drug screening ^1^. For these devices to perform in an optimal manner, a high level of control over the immobilization process is crucial (see Figure 1). In the case of biosensors, such control will ensure that the analyte binding site of a receptor molecule is optimally accessible and a device with maximum sensitivity is generated. Similarly, biocatalysts are preferably immobilized in a directed fashion such that their active site can easily be reached by substrate molecules and, hence, maximum activity is obtained. Control over the immobilization process also contributes to the homogeneity of the surface coverage, such that devices are generated with uniform and well-defined characteristics, providing reproducible and accurate output ^2^.

In the past few decades, researchers have devoted substantial effort to developing new strategies for site-specific protein immobilization. Non-covalent approaches, also referred to as affinity-mediated immobilization, include the use of protein A or G for binding of antibodies, peptide tags such as polyhistidine, protein tags such as maltose-binding protein and glutathione- *S*-transferase, DNA-directed immobilization, and the biotin–streptavidin interaction pair. While highly useful, these approaches will not be discussed in this review. Instead, the reader is referred to excellent reviews published elsewhere ^2– 7^.

The present review focuses on covalent methods for site-specific protein immobilization. Covalent immobilization provides a distinct, more robust, and stable way of tethering proteins to a surface. Such modification of surfaces becomes important where more permanent properties are required, e.g. in medical sensors and implants. To a large extent, the advances made in this field represent implementations of developments in the more general field of site-specific protein modification. These developments comprise the discovery of new bioorthogonal reactions that proceed under physiological conditions between chemical groups that are absent in, and do not cross-react with, endogenous functionalities in proteins ^8, 9^. In addition, they include methods for the site-specific introduction of these bioorthogonal groups into proteins ^10^. Worth mentioning, and potentially more appealing to researchers with a medical or biological background, are also the advances in the field of enzyme-mediated protein modification ^11, 12^.

Broadly speaking, we will highlight a selection of both enzyme-mediated and chemical approaches for covalent, site-specific protein immobilization. Illustrative examples from the past five years will be provided with the intention of demonstrating the intriguing diversity regarding the nature of the proteins, solid supports, and application purposes for which these approaches have been employed.

## Enzymatic approaches for covalent, site-specific protein immobilization

### Enzyme-mediated immobilization: sortase A

**Figure 2.**
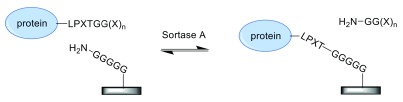
Sortase-mediated protein immobilization. Instead of pentaglycine, the solid support can be modified with substrate nucleophiles consisting of a smaller number of glycine residues. Alternatively, the LPXTG recognition sequence is coupled to the surface, and the protein of interest is tagged with an N-terminal oligoglycine motif.

Sortase A is a transpeptidase from the Gram-positive bacterium *Staphylococcus aureus* that recognizes a LPXTG sequence at the C-terminus of a target protein. Using a cysteine thiol nucleophile, it cleaves between the T and G residues of this sequence, yielding an acyl-enzyme intermediate. Subsequently, an N-terminal pentaglycine amine nucleophile attacks the thioester to complete the ligation reaction. The use of sortase for protein modification was introduced in 2007 ^13, 14^. Since then, the approach has been exploited for a range of biotechnology applications including protein immobilization (see Figure 2) ^15^.

Recent examples of the use of sortase for site-specific protein immobilization include the conjugation of adhesion proteins to fluorescent microsphere beads ^16^, the modification of liposomes with green fluorescent protein (GFP) ^17^, the production of an influenza virus protein array on glass slides ^18^, antibody and enzyme immobilization on cellulose nanocrystals ^19^, immobilization of a bait protein to agarose beads for application in affinity purification mass spectrometry ^20^, and layer-by-layer immobilization of two fluorescent proteins on gold ^21^. An interesting aspect of the last-mentioned study is the fact that two sortase variants were used with orthogonal substrate specificities. This facilitated the immobilization of GFP as the second protein layer on top of a layer of immobilized red fluorescent protein.

Sortase-mediated reactions reach a dynamic equilibrium because the reaction product is also a substrate for the enzyme. As a consequence, sortase-mediated reactions have low efficiency, and a large excess of both enzyme and substrate is required to obtain sufficient conversion. The reaction efficiency has been improved in different ways, amongst others by using a β-hairpin structure around the ligation site ^22^ or depsipeptide substrates ^23^. Both strategies prevent the reversible reaction from occurring. In addition, sortase variants with increased ligation activity have been evolved ^24^. Of particular interest in this context is the work by Heck *et al*. in which the authors developed an assay to follow the sortase-mediated ligation on microparticles by real-time flow cytometry ^25^. This assay allows for the comparison of different enzymatic (and chemical) immobilization strategies and the optimization of reaction parameters for enhanced immobilization performance.

The reversibility of the sortase-mediated reaction can be advantageous in applications in which the protein is denatured or deactivated over time. In such a case, sortase can be used for the regeneration of the bioactive surface, as demonstrated by Ham *et al.* ^26^. In addition, the reversibility of the reaction allows for the assessment of the degree of immobilization by cleaving the previously tethered protein followed by quantification using well-established solution-phase techniques. This approach was employed by Cambria *et al.* to determine the amount of immobilized human epidermal growth factor on functionalized hydrogels ^27^.

### Immobilization by an enzyme self-labeling tag: the SNAP-tag

**Figure 3.**
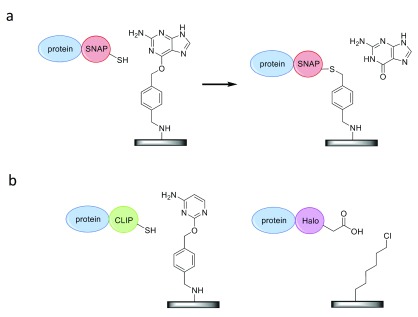
Site-specific protein immobilization by fusion to self-labeling tags. ( **a**) The SNAP-tag, 20 kDa in size, reacts with *O* ^6^-benzylguanine. ( **b**) The CLIP-tag (also 20 kDa) reacts with *O* ^2^-benzylcytosine substrates, whereas the HaloTag (33 kDa) forms a covalent bond through nucleophilic displacement of halides from alkyl halide substrates.

The SNAP-tag, which was first reported in 2003 by Keppler *et al*., is one of the most versatile self-labeling protein tags ^28^. The protein, which is a mutant version of the human DNA repair protein *O* ^6^-alkylguanine-DNA-alkyltransferase (AGT), reacts with benzylguanine derivatives via an internal reactive cysteine residue, thereby forming a covalent thioether bond upon the release of guanine. In order to facilitate covalent, oriented protein immobilization, the SNAP-tag, which is 20 kDa in size, is fused to a target protein, while the solid support is functionalized with a benzylguanine derivative (see Figure 3A) ^29^.

In the past few years, the technology has been employed for the immobilization of GFP and extracellular fragments of the adhesion protein E-cadherin on gold surfaces containing a self-assembled monolayer of benzylguanine thiol mixed with methoxy-capped tri(ethylene glycol) undecanethiol ^30, 31^. Variation of the benzylguanine thiol concentration allowed for control over the protein density. The method was applied to investigate the adhesive functionality of E-cadherin surfaces by adhesion force spectroscopy. In a second example, SNAP-tagged fluorescent proteins and cadherin were coupled to quantum dots coated with amino-poly(ethylene glycol) and functionalized with an *N*-Hydroxysuccinimide ester derivative of benzylguanine ^32^. The fluorescent properties of the quantum dots and the model protein GFP enabled estimation of the immobilization efficiency. Recker *et al.* employed the SNAP-tag technology for the immobilization of cytokines on polystyrene particles ^33^. The directionally immobilized cytokines were shown to be fully signaling competent in cell culture, supporting the potential use of this approach for basic research on cytokine signal transduction and the improvement of biomaterials through functionalization with cytokines.

Additional examples of self-labeling protein tags include the HaloTag (33 kDa) ^34^ and the engineered variant of the SNAP-tag, CLIP-tag (see Figure 3B) ^35^. Interestingly, the orthogonality in substrate specificity ( *O* ^2^-benzylcytosine vs. *O* ^6^-benzylguanine) allows for simultaneous and specific reaction of SNAP and CLIP fusion proteins with different molecular probes or, potentially, with positional control of immobilization on the same substrate surface.

## Chemical approaches for covalent, site-specific protein immobilization

In the past few decades, several bioorthogonal chemistries have been developed for site-specific labeling of proteins and other biomolecules ^8, 9^. The development of these chemical reactions has greatly been driven by the desire to selectively label a target molecule in complex mixtures of biomolecules, such as cells or living organisms. In the following section, it will be shown how these bioorthogonal reactions can be very useful for the ligation of proteins in chemically less complicated media (that is, for immobilization purposes) as well.

**Figure 4.**
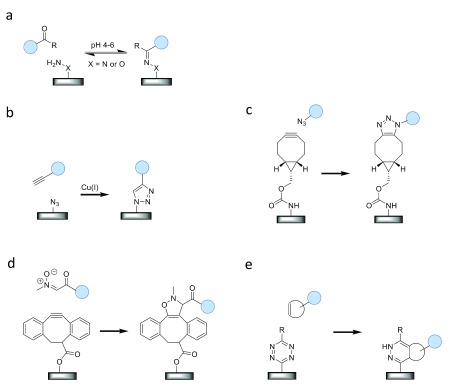
Selection of chemical approaches for covalent, site-specific immobilization of proteins. ( **a**) Oxime ligation, ( **b**) Cu(I)-catalyzed azide–alkyne cycloaddition (CuAAC) reaction, ( **c**) strain-promoted azide–alkyne cycloaddition (SPAAC) reaction, ( **d**) strain-promoted alkyne–nitrone cycloaddition (SPANC) reaction, and ( **e**) inverse electron-demand Diels–Alder reaction (IEDDA) reaction.

The site-specific nature of these approaches depends on the fact that the functional groups which are employed in the different reactions are absent and do not cross-react with endogenous amino acids. Different site-specific methods can be used to introduce these groups into the protein of interest, after which the chemical immobilization reaction can occur. What is noteworthy is that the same method (for example, enzyme-mediated modification) has been used to introduce different functionalities (such as aldehydes, azides, or alkynes). This section is not meant to be exhaustive but rather describes a selection that, in our opinion, clearly demonstrates the value of the best chemical approaches for protein immobilization. A description of additional approaches, such as native chemical ligation, the Staudinger ligation, thiol-ene chemistry, photochemistry, and more specifically the combination of non-covalent interaction with photochemistry, can be found elsewhere ^1, 36– 39^. An overview of the five elegant approaches discussed here is depicted in Figure 4.

### Oxime ligation

The condensation of aldehydes or ketones with aminooxy or hydrazide compounds to give a stable oxime or hydrazone linkage, respectively, is known as the oxime ligation. The reaction is generally slow at neutral pH and is normally performed at pH 4–6. The fact that neither the ketone/aldehyde nor the aminooxy/hydrazide is naturally present in proteins makes this reaction ideal for oriented protein immobilization.

Ketones or aldehydes can be introduced into proteins in a site-specific manner through the incorporation of non-natural amino acids, expressed protein ligation (EPL), or enzyme-mediated strategies ^8^. Additionally, the aldehyde or ketone moiety can be introduced by N-terminal transamination, by periodate oxidation of an N-terminal serine or threonine residue, or by periodate oxidation ^40^ or metabolic labeling ^41^ of glycans in antibodies and other glycoproteins. Importantly, a recent advancement with respect to the oxime ligation (a reaction which itself has been known for decades) is the discovery that aniline can be used as a catalyst ^42^. It allows the reaction to be performed at neutral pH, making it useful for pH-sensitive proteins as well. However, it may be noted that oxime ligations are carried out under reversible conditions and although equilibrium is in strong favor of the product, oximes are prone to slow exchange reactions and hydrolysis. Therefore, in situations requiring high stability, the oxime or hydrazone bond is typically reduced with sodium cyanoborohydride.

Cho *et al*. reported one of the latest examples utilizing the oxime ligation for oriented protein immobilization *.* Two enzymes, alkaline phosphatase and methyltryptophan oxidase, were tethered to amine-coated beads and similarly coated surfaces of a 96-well plate ^43^. In order to introduce the aldehyde moiety into these model proteins, the researchers utilized formylglycine-generating enzyme, which is known to oxidize the cysteine of a six-amino-acid recognition sequence. This ligation was stabilized by oxime reduction ^44^.

In other applications, however, the reversible nature of the oxime ligation can be exploited. Rashidian *et al*. describe the chemoenzymatic, reversible immobilization of GFP and the therapeutic protein glucose-dependent insulinotropic polypeptide. Protein farnesyl transferase (PFTase) was used for the introduction of the aldehyde moiety ^45^. Interestingly, the proteins, containing a C-terminal tetrapeptide as the PFTase recognition sequence, could be captured selectively from a crude cellular extract. After coupling to hydrazide-modified agarose beads, the proteins were released under mild conditions by the addition of an alkoxyamine. The use of synthetically modified alkoxyamines, containing a fluorophore or a PEG chain, allowed for the simultaneous modification of the purified proteins.

### Azide–alkyne cycloaddition

The 1,3-dipolar cycloaddition between azides and alkynes is one of the best-known click reactions ^46– 48^. Essential for its popularity has been the discovery by both the Meldal and Sharpless research groups that the reaction proceeds at room temperature if Cu(I) is used as the catalyst ^49– 51^. The reaction has found widespread use in a myriad of applications, including the investigation and manipulation of proteins ^52– 55^. Also for protein immobilization, the Cu(I)-catalyzed azide–alkyne cycloaddition (CuAAC) and its strain-promoted variant (SPAAC) ^56^ have become more and more popular. Like aldehydes and ketones, azides and alkynes are not naturally present in proteins and thus have to be introduced using either a biosynthetic or a chemical approach.

The incorporation of non-natural amino acids, in particular, has been shown to be an attractive approach. Both site-specific introduction using the amber suppression method ^57^ and the more straightforward approach of residue-specific replacement ^58^ have been employed for this purpose. For example, an engineered variant of GFP, functionalized with a single azide by expression in an auxotrophic bacteria strain using azidonorleucine as a methionine surrogate, was immobilized on SPR sensor surfaces via the SPAAC reaction ^59^. The same protein, site-specifically functionalized via genetic encoding of a cyclooctyne-containing amino acid, was used to demonstrate the use of the SPAAC reaction in biomolecular patterning of glass surfaces ^60^. In this proof-of-concept study, the increase in fluorescence of azidocoumarin-modified glass upon reaction with a cyclooctyne provided a convenient read-out of the immobilization process.

GFP also acted as the model protein in an interesting study in which the effect of the type of solid support, the linker length, and the immobilization site on CuAAC-mediated immobilization efficiency was investigated ^61^. For this purpose, the amber suppression method was utilized to facilitate the introduction of *para*-azidophenylalanine at three different positions in the protein. Polystyrene, TentaGel – consisting of poly(styrene-oxyethylene) graft copolymer – and Sepharose (cross-linked agarose) resins were selected as solid supports. Sepharose derivatized with propargyl alcohol afforded the highest yield of immobilization. Up to 2-fold difference in immobilization efficiency was observed depending on the location of the clickable handle.

Trilling *et al.* combined non-natural amino acid incorporation with the SPAAC reaction for oriented immobilization of antibody fragments ^62^. The variable domain of a llama antibody was functionalized with a single azidohomoalanine residue and coupled to a bicyclononyne-modified SPR chip. It was shown that the oriented immobilization led to a strongly increased sensitivity of the biosensor when compared to an SPR chip containing randomly immobilized antibodies. More recently, Lühmann *et al*. demonstrated the site-specific conjugation of fibroblast growth factor 2 to agarose beads through genetic encoding of propargyl-lysine followed by the CuAAC reaction ^63^. The immobilized growth factor was shown to have retained its ability to induce cell proliferation. Of interest for the field of biocatalysis is the study reported by Wu *et al*. The model enzyme T4 lysozyme was immobilized on superparamagnetic beads ^64^. Site-specific, CuAAC-mediated immobilization enhanced the stability of the enzymes towards freeze-thaw cycles and the denaturant urea when compared to enzyme immobilized in a random manner on epoxy-modified beads. The activity of the immobilized enzyme was shown to vary depending on the site of conjugation.

Instead of non-natural amino acid incorporation, EPL can facilitate N- or C-terminus-specific functionalization of proteins with azides or alkyne moieties ^65– 67^. The method has been combined with the azide–alkyne cycloaddition, amongst others, to immobilize GFP and aldo-keto reductase on a PEG-based solid support ^68^, GFP and single-chain variable fragment antibodies on agarose beads ^69^, and nanobodies on polymersome vesicles ^70^. A HER2 affibody, a 6.5 kDa protein with high affinity for the oncogene HER2/neu receptor, has been tethered to superparamagnetic iron oxide (SPIO) nanoparticles ^71^. This approach was used to target the nanoparticles, acting as imaging agents, to tumor cells. What is noteworthy is that these HER2–SPIO nanoparticles, obtained by the combination of EPL and CuAAC chemistry, were shown to perform better than SPIO nanoparticles to which the same affibody was conjugated using random immobilization approaches.

As a third approach, enzymes have been employed for the introduction of azides and alkynes into proteins. For example, Keillor and colleagues showed that microbial transglutaminase is able to catalyze the attachment of propargyl amine to the C-terminus of proteins ^72^. A heptapeptide, also called Q-tag, functioned as the recognition sequence. In a proof-of-principle experiment, a Q-tagged fluorescent protein was immobilized to magnetic azide-modified nanoparticles using the CuAAC reaction as the conjugation method. Moreover, Qu *et al*. combined enzymatic modification with the SPAAC reaction for the immobilization of thrombomodulin ^73^. Sortase A was employed to introduce an azide moiety at the C-terminus of this blood-coagulation-reducing protein. Subsequently, the protein was tethered to dibenzocyclooctyne-modified vascular grafts, which were thus rendered thromboresistant. Interestingly, it was shown that the dibenzocyclooctyne-modified surfaces could be sterilized by ethylene oxide prior to the strain-promoted [3+2] cycloaddition. In this way, the described strategy has great potential for clinical application by providing a sterile off-the-shelf product ready to be coated with azide-modified therapeutic protein right before implantation.

As a chemical alternative to the abovementioned biosynthetic methods, we recently showed that a single azide can be site-specifically introduced at the N-terminus of GFP using the pH-controlled and metal-free diazo transfer reaction ^74^. This straightforward procedure was combined with the SPAAC reaction to immobilize azido-functionalized GFP on bicyclooctyne-modified vertical GaAs nanowires ^75^. It was shown by fluorescence microscopy that anti-GFP antibody bound to the immobilized protein. The experiment functioned as a proof-of-concept study, demonstrating the feasibility of functionalizing vertical semiconductor nanowires for biological applications, such as biosensing and the study of protein–protein interactions.

### Other click reactions

In 2010, the need for a bioorthogonal reaction faster than SPAAC for the labeling of proteins in living systems led to the development of the strain-promoted alkyne–nitrone cycloaddition (SPANC) ^76^. The 1,3-dipole nitrone is used as a more reactive alternative for an azide, leading to reaction rates that are up to 30 times faster. Despite the fact that its use for protein labeling in general has been limited so far, the reaction has already been successfully applied for protein immobilization. Single-chain variable fragment antibodies against the tumor marker HER2 were coupled to fluorescent, superparamagnetic nanoparticles ^77^. The nitrone group was introduced specifically at the N-terminus of the antibody molecules. Binding of the targeted nanoparticles to HER2-positive cells was observed, demonstrating the feasibility of the approach and its potential use as a general strategy for the development of a new generation of targeted nanoparticles.

The inverse electron-demand Diels–Alder reaction (IEDDA) between 1,2,4,5-tetrazines and strained alkenes or alkynes is another click reaction that was recently introduced ^78^. Especially, when *trans*-cyclooctene is used as the strained alkene, the reaction rate is extremely high, exceeding those of the SPAAC, SPANC, and CuAAC reactions ^79^. However, speed and concomitantly lower usable reagent concentrations are typically of less importance in the case of protein immobilization. Researchers have, nevertheless, explored the use of the IEDDA reaction for this application ^80– 82^. So far, though, the method has been used only in a non-specific manner by functionalizing the proteins of interest with an NHS-derivative of tetrazine or *trans*-cyclooctene. Non-natural amino acids bearing various strained alkenes, alkynes, and tetrazine have been synthesized and can now be incorporated into proteins in a site-specific manner ^83– 85^. Therefore, it is expected that the first reports employing the IEDDA reaction for oriented protein immobilization will appear in the near future.

## Concluding remarks

In conclusion, we believe that the large set of methods available today to site-specifically immobilize proteins to solid supports holds great promise for future use in applications ranging from biosensor development to biocatalyst immobilization and next-generation biomaterials. A considerable number of examples performed on a diverse set of proteins and solid supports has been provided that substantiate this prospect. Particularly exciting are those studies that are close to a final application, such as the thrombomodulin-modified vascular grafts ^73^. An important note is that several more or fewer orthogonal click reactions have appeared over recent years that in years to come will allow us to carry out macrostructured immobilization of a range of functional proteins working in concert.

Unfortunately, most reports deal with proof-of-concept studies and only in a few cases has the immobilization efficiency been addressed. Therefore, it is currently difficult to compare the different approaches and to select the optimal method for a specific application. In our opinion, it would be extremely interesting if more analytical studies would be performed such as the one reported by Heck *et al*., comparing the ligation efficiency of several sortase variants ^25^. Of interest is also the kind of analysis performed by Lühmann *et al*. ^63^, in which the immobilization efficiency was estimated as the fraction of bound protein relative to the total amount of protein used. The more common measure of expressing the yield as the fraction of surface that got covered ^20, 32, 33^ does not provide any information on the amount of protein that was lost during the immobilization process. This information will be valuable from an economical point of view and when protein availability is limited, for example because of low expression yields, often associated with less stable and more interesting proteins. It should be noted that regarding the surface coverage, it is the fraction of *active* protein molecules that is most relevant. When proteins other than fluorescent proteins are immobilized, such analysis will obviously be more challenging. The control of surface density is another aspect that has received little attention so far. This feature can be controlled in the primary functionalization step in which the surface is efficiently and stably modified with, for example, azide or alkyne groups for the CuAAC or SPAAC reaction ^75^ or pentaglycine or benzylguanine for sortase- or SNAP-tag-mediated protein immobilization, respectively ^15, 29^. As described above, Fichtner *et al*. used such an approach to vary the density of adhesion protein on gold surfaces ^31^.

Undoubtedly, it is not only the efficiency of the immobilization strategy that plays a role in the selection process. Another decisive factor is the freedom in selecting the site of conjugation, which is highest in the case of the chemical approaches combining non-natural amino acid incorporation with one of the click reactions. Low stability and sensitivity of the protein to low pH or towards an organic or metal catalyst will point to one of the newest click reactions such as SPAAC, SPANC, and IEDDA. However, one should be aware of the hydrophobicity of the functionalities involved in these reactions, which may lead to incompatibilities and inefficient conjugation ^86^. Expression yields are generally lower when non-natural amino acids are incorporated. While studies are ongoing to improve these yields (as exemplified by the work of Schmied *et al.* ^87^), this fact supports the relevance of measuring the fraction of immobilized protein, i.e. the yield of reaction (loss of protein) at a given protein concentration.

For researchers with limited expertise regarding synthesis and surface modification with, for example, strained alkynes and tetrazines, enzymatic strategies will often be appealing. An important advantage of the last-mentioned methods is the fact that the protein can be produced under native conditions, providing lower costs and higher yields compared to the strategies involving non-natural amino acid incorporation. In such ligations, however, an enzyme recognition sequence or enzyme tag will always need to be added to the protein.
